# Tickling in the Throat

**Published:** 1869-01

**Authors:** 


### HALL'S

### JOURNAL OF HEALTH.

NEW SERIES.

VOL. I. JANUARY, 1869. NO. 1.

### TICKLING IN THE THROAT

Always precedes death by common consumption of the lungs in about two years, on an average; but this tickling is not always followed by consumption; common prudence then suggests that in every case of tickling in the throat, which seems to be at the little hollow at the lower part of the windpipe, or just above the top of the breast-bone—especially if such a sensation is more or less decided for days and weeks, at intervals—an effort should be made to ascertain its character, and to use safe and judicious means for its removal.

It would not be safe practice to use means to destroy the sensation of tickling, merely smothering the symptom, while the cause of it was in operation. Shutting up the hatches of a ship, to prevent the escape of smoke, while the hold is on fire, does not quench the flame, though it may seem to some to do so.

It would not be judicious to apply a remedy to the tickling spot when the cause of the sensation was a foot or two away in a different organ of the body. Both these positions will be better understood by enumerating some of the causes of the tickling in the throat:

1. If a man laughs heartily there will sometimes be such a hasty, urgent tickling as to make the cessation of laughter imperative.

2. Persons in robust health will cough violently on retiring to bed, the tickling being occasioned by lying with the hands or arms uncovered, thus cooling the skin, contracting the pores, and driving in upon the lungs, to oppress and irritate, what should more naturally have had an exit from the body in the shape of insensible perspiration through the skin; as soon as the arms are covered up and have had time to become healthfully warm on the surface, the tickling ceases and the cough disappears.

Suppose that paregoric, laudanum, or any other of the thousand and one remedies for coughs, colds, and consumption should be given to an extent sufficient to dull the sensation of tickling, as any anodyne would do, the cough would cease for awhile, but the cause being in operation, the skin would become colder and colder, tending to produce before the morning an attack of pleurisy, lung fever, or dangerous hemorrhage.

3. Many a person has gone to bed at night to be waked up in a few minutes with a cough, which would continue for hours, most effectually preventing sleep; irritating the mind, making the body more and more restless; the cough meanwhile growing more and more annoying until there is first gagging, and finally vomiting of everything eaten at the last meal, and in ten minutes the person will be sound asleep and remain so until the morning. On inquiry, it will be found that the food was thrown up almost unchanged, except that it was "as sour as vinegar;" in other words, it was undigested. The person had eaten too much, or too fast, or had taken something which the stomach could not work up.

Suppose an anodyne, or a "trochee," or a "tablet" had been taken, to an extent sufficient to remove the tickling or to smother the cough; the undigested food which caused the tickling would have remained in the stomach, becoming more and more sour and noxious, until nature, outraged, brings spasms, convulsions, or apoplexy to relieve herself. This is a

STOMACH COUGH,

Requiring the removal of its contents by an emetic, and not such remedies as would merely smother up the sensation of tickling, which, although felt at the bottom of the throat, was caused by a certain condition of things in the stomach a foot or two distant; much in the same manner as the tingling or numbness is felt at the ends of the fingers sometimes, when a blow has been given at the elbow. There is a nerve with two branches, one of which goes to the stomach, the other to the throat and lungs, and in certain conditions, when one branch is in a suffering condition, the other is more or less affected; and this is what physicians mean sometimes when they speak of the relative condition of two parts as being in "sympathy."

4. Many have experienced the sensation and know very well the meaning of the expression which we very often hear of a

CRUMB GOING THE WRONG WAY,

Induced by a particle of food or drop of water going into the windpipe and down to the top of the lungs, instead of being passed into the stomach; this misdirection being occasioned by attempting to breathe at the instant of swallowing; in this case, nature sets up a violent and irrepressible tickling in the throat, the object of which is to excite cough, which is a violent expulsion of air through the branches of the windpipe down among the lungs, and through the windpipe itself, in the hope, as it were, that the offending particle may be thrown out of the system; here nature originates her own mode of cure—excites a cough. Sometimes the particle is so large that the cough cannot dislodge it; then the surgeon must cut down into the lungs and take it out; otherwise the cough would become so violent as to cause fatal hemorrhage of the lungs, by the bursting of a large blood-vessel, from the strain of the violent coughing.

But suppose a remedy had been addressed to the tickling sensation, and had suppressed it, which means removing the cough, then the foreign matter would remain in the lungs, to cause in a few days a dangerous or even fatal form of inflammation or of pneumonia.

CROUP

Is a word of terror to every young mother; it is simply a form of diphtheria—the very sound of which is often the knell of death. This croup is instantly known by the peculiarity of the cough, which once heard by a parent will never be forgotten. The essence of the disease is the formation of a solid substance on what is commonly spoken of as the inner side of the windpipe; this goes on thickening until the windpipe is closed and air enough cannot pass into the lungs to support life, and the poor sufferer is smothered to death. The true remedy is to do something which will loosen or detach and absorb this membrane, allowing the tickling and cough to remain, so as to force it out of the windpipe as soon as it is loosened; it often comes out a mass of almost leathery tenacity. This being the case, as every intelligent physician will admit, whatever is done to remove the tickling, or the cough, does just that much towards destroying all chance of life.

In all the above cases, the tickling in the throat is nature's mode of exciting cough, and it is precisely so in consumption.

COUGH IS CURATIVE!

Is nature's cure, and to smother cough without removing what causes it, is to hinder nature and take away all chance of cure. When a man clearly has consumption, coughs a great deal, has been bringing up yellow matter for a long time, if his cough should subside he will inevitably die in three or four days, because the cough helps to bring that matter out of the lungs and keeps them clear; but when the cough becomes so weak or so unfrequent as not to remove the matter as fast as it is formed, the lungs begin to fill up with it, air cannot get in, and life ends. The only hope of curing consumption is to promote cough on the one hand, so as to get the lungs clear of the matter in them, and prevent the formation of more. But the popular sentiment is, that in proportion as there is less cough, the chances of life are increasing, and willingly and hopefully the patient takes what "cures his cough," and is thus led a willing victim to the grave of his own digging. So much are men, with all their boasted intelligence, like the silly creature which feels itself safe when it can hide its head in a hole, to be crushed the next instant in the jaws of its relentless pursuer.

